# Dissected saccular aneurysms of the ascending aorta

**DOI:** 10.1002/ccr3.2506

**Published:** 2019-10-27

**Authors:** Hazem Aljasem, Mohammad Bashar Izzat

**Affiliations:** ^1^ Department of Surgery Faculty of Medicine Damascus University Damascus Syrian Arab Republic

**Keywords:** aneurysm, aorta, hemoptysis, pathology, surgery

## Abstract

This report highlights the need for distinction between saccular and fusiform aortic aneurysms, considering the high risk of rupture of saccular aneurysms. The management of dissected saccular aneurysms involves elective replacement of the dissected aorta while preserving the aortic valve.

## Graphical abstract

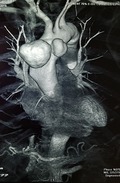

## 1 INTRODUCTION

Dissected saccular aneurysms of the aorta are very rare. They tend to develop in areas of healed microscopic dissection of the aortic wall and are thought to be more prone to rupture compared to fusiform aneurysms,^1^, ^2^ and hence, they mandate urgent surgical attention.^3^ Here we present two cases of dissected saccular aneurysm of the ascending aorta which were managed by surgical replacement of the diseased ascending aorta.

## 2 CASE REPORTS

### 2.1 Case 1

**Figure 1:**
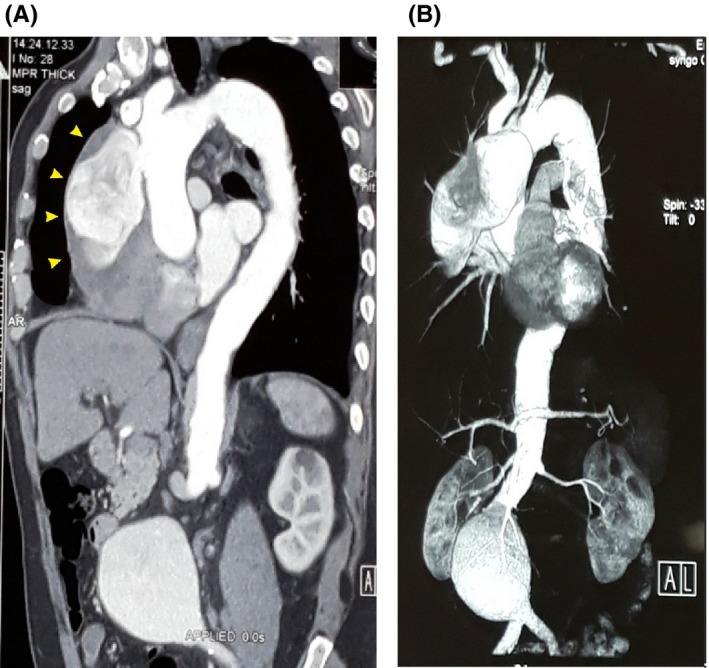
A, Contrast‐enhanced CT scanning and B, CT angiography of the chest and abdomen in case 1. A large saccular aneurysm of the ascending aorta (yellow arrowheads) and a fusiform aneurysm of the abdominal aorta are shown

A previously healthy 68‐year‐old man presented with recurrent cough productive to blood‐streaked sputum. Chest X‐ray showed marked widening of the upper mediastinum, and echocardiography revealed a huge aneurysm of the ascending aorta, with an intact aortic valve and normal left ventricular function. Contrast‐enhanced CT scan and CT angiography of the chest and abdomen were performed and demonstrated a large (96 × 56 × 58 mm) saccular aneurysm of the ascending aorta, in addition to a 96 × 57 × 59 cm abdominal aortic aneurysm (Figure 1). Bronchoscopy and coronary angiography did not reveal any abnormalities.

During surgery, a large saccular aortic aneurysm was found 3.5 cm above the aortic valve. Under hypothermic circulatory arrest condition, transecting the aorta revealed a shelf‐like protrusion of the aortic wall at the proximal edge of the aneurysm. The ascending aorta per se was mildly atherosclerotic, with a wide shallow depression proximal to the lesion without gross dilatation or dissection. The entire aneurysmal wall was excised, and multiple fistulae were noted between the posterior aneurysmal wall and the lower lobe of the right lung, and were closed directly using 3‐0 Vicryl sutures. The ascending aorta was replaced with a 32 mm Dacron vascular graft. Postoperative course was uneventful, and the patient was referred to the vascular surgeon to manage the abdominal aneurysm.

### 2.2 Case 2

**Figure 2:**
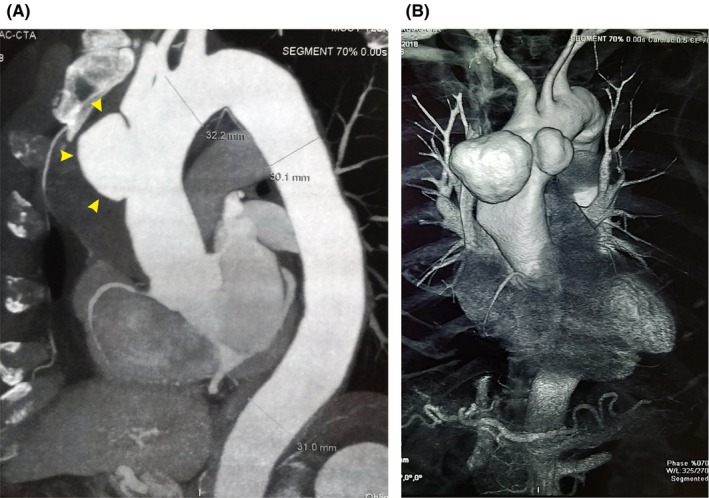
A, Contrast‐enhanced CT scanning and B, CT angiography of the chest in case 2. A mushroom‐shaped aneurysm of the ascending aorta is shown (yellow arrowheads)

A 59‐year‐old male patient with a history of chronic hypertension was referred to our hospital with vague chest discomfort. Echocardiography confirmed the presence of a large ascending aortic aneurysm, with trace aortic valve incompetence and preserved left ventricular function. CT angiography of the chest demonstrated two mushroom‐shaped aneurysms of the ascending aorta, measuring 57 × 51 × 39 mm and 42 × 37 × 39 mm (Figure 2). Coronary angiography did not reveal any coronary irregularities.

Intraoperatively, under hypothermic circulatory arrest condition, two saccular aneurysms were noted in the ascending aorta, 40 mm distal to the sino‐tubular junction, with shelf‐like protrusions at both the proximal and distal edges of the lesions. Both lesions had thrombi in the proximal aspects of their lumens, and the ascending aorta per se was moderately to severely diseased, with multiple atherosclerotic ulcerations. The entire diseased wall of the ascending aorta was excised and replaced with a 30 mm Dacron graft. Postoperative course was uneventful.

## 3 DISCUSSION

Dissected saccular aneurysms of the aorta are extremely rare and are thought to be associated with a higher risk of rupture compared to their fusiform counterparts.^2^ This entity has been observed to develop in areas of “healed microscopic dissection” of the aortic wall, which were defined as zonal degeneration and fibrosis extending throughout the intima and the middle third of the media.^3^ This process involves ulceration of the intima followed by aneurysm formation. Characteristically, edges of dissected saccular aneurysms protrude like shelves, which consist histologically of the inner part of the dissected media, and are associated with medial thinning at the center of the aneurysmal wall.^3^, ^4^ These lesions differ from organized thrombi in the false lumens of conventional aortic dissections in that intramural hemorrhages or thrombi are absent.^4^

To the best of our knowledge, only few cases of dissected saccular aneurysms of the aorta have been described. We reported here two cases of huge dissected saccular aneurysms of the ascending aorta that underwent surgery at our service. The first patient presented with hemoptysis as a result of fistulous formation, and this has not been reported before. Due to the anatomical locations of the aneurysms and their close proximity to posterior sternal plates, establishing cardiopulmonary bypass before opening the chest was mandatory. The right subclavian artery was selected for arterial input in the first case due to the presence of a large abdominal aneurysm, while the right femoral artery was used in the second patient. Aortic valve cusp morphology and function were normal in both patients; therefore, both cases were managed successfully with replacing the diseased ascending aorta using Dacron grafts while preserving the aortic valves. Our two cases should draw attention to the satisfactory outcome of elective surgical repair of dissected saccular aneurysms of the aorta.

## CONFLICT OF INTEREST

None declared.

## AUTHOR CONTRIBUTIONS

Hazem Aljasem and Mohammad Bashar Izzat: conceived and acquired the data, drafted the manuscript and revised it critically, and gave the final approval of the version to be published.

## ETHICAL APPROVAL

All procedures performed in this study were in accordance with the ethical standards of the Damascus University Research Ethics Committee and with the 1964 Helsinki declaration and its later amendments.

## INFORMED CONSENT

Informed consent was obtained from all individual participants included in the study.
